# Challenges and possible solutions to peritoneal dialysis use in Nigeria

**DOI:** 10.11604/pamj.2020.35.138.21066

**Published:** 2020-04-28

**Authors:** Samuel Ajayi, Yemi Raji, Temitope Bello, Ayodeji Arije

**Affiliations:** 1 Department of Medicine, College of Medicine, University of Ibadan, Ibadan, Oyo State, Nigeria; 2 University College Hospital, Ibadan, Oyo State, Nigeria

**Keywords:** Peritoneal dialysis, challenges, solutions, Nigeria

## Abstract

**Introduction:**

Peritoneal dialysis is a form of renal replacement therapy that is both effective and relatively affordable. Peritoneal dialysis (PD) was first used in Nigeria as a treatment option for renal failure. Its use was first reported in Nigeria in 1969 and became more widespread in the 80s and 90s. Haemodialysis, which is capital intensive to set up and requires infrastructures and facilities such as electricity, intense water consumption and buildings, seems to have upstaged peritoneal dialysis both in demand and supply.

**Methods:**

This cross-sectional study is a convenient survey of nephrologists, renal technicians and nurses in Nigeria. We used a structured, self-administered questionnaire on a cross-section of members and associate members attending a national nephrology association meeting.

**Results:**

There were 68(54.4%) doctors, 43(27.2%) nurses, and 14(11.2%) renal technicians, all from medical institutions with renal treatment programs who participated in the study. The most common problems encountered with PD use are financial constraints (51.7%), inadequate fluid supply (50%), frequent line blockage (22.4%) and frequent infections (17.2%). Reasons attributed to the stoppage of PD in the centres included lack of PD fluids (50.8%), unavailability of PD catheters (22.8%), lack of expert personnel to train (15.8%).

**Conclusion:**

Main challenges to peritoneal dialysis use in Nigeria include limited experience and training and availability and cost of consumables. Effort to overcome the factors militating against its use should be positively pursued so that peritoneal dialysis will be re-integrated into the mainstream of renal replacement therapy once more.

## Introduction

Peritoneal dialysis is a form of renal replacement therapy that is both effective and relatively affordable. It is especially useful in patients who have hemodynamic instability in whom haemodialysis may be a challenge. Peritoneal dialysis (PD) was first used in Nigeria in as a treatment option for renal failure. Its use was first reported in Nigeria in 1967 [1] and became more widespread in the 80s and 90s. Mostly used for acute kidney injury in the past, indications soon included treating complications of chronic kidney disease and poisoning PD was ideally suited for treating children with renal failure [2-7], in whom vascular access could be problematic. The most utilized form of peritoneal dialysis was the intermittent form for acute dialysis, usually for patients with acute renal failure. It was easy to do and was usually done in the hospital. Added to this was the fact that it was relatively cheap because there were many vendors marketing consumables. Indeed, in Nigeria peritoneal dialysis and catheter fluids were being manufactured and widely distributed in the past.

Surprisingly, haemodialysis, which is capital intensive to set up and requires infrastructures and facilities such as electricity, intense water consumption and buildings, seems to have upstaged peritoneal dialysis both in demand and supply. In consequence of all these, there was failing interest in marketing peritoneal dialysis consumables, leading to difficulty in procuring PD fluids. In addition, several medical problems associated with its use have made it less popular, the most important being frequent peritoneal infection [3]. Therefore, although we have as many as 30 renal centres in Nigeria, there are only very few centres offering peritoneal dialysis services. Indeed, recent observation suggests its use has dwindled dramatically in the last ten years. This is not happening only in Nigeria, but across Africa in general where it has recently been noted that PD prevalence is 2.2 per million population (pmp) compared with global prevalence of 27pmp [8] and even then 85% of African PD population is in South Africa [8]. This study was undertaken to explore the challenges and possible solutions to the use of peritoneal dialysis in Nigeria. Specific objectives of the study include: assessment of the use of PD for renal failure in Nigeria, identify the possible factors and challenges responsible for the dwindling use, and define the perception of renal health practitioners on the role of PD in renal care with a view to offering solutions for these challenges. A good way forward would be to hear directly from renal care practitioners themselves.

## Methods

This cross-sectional study is a convenient survey of nephrologists, renal technicians and nurses in Nigeria. The advantage of this convenient sampling method [9] was that all professional groupings required for this kind of study were available to participate. This would otherwise have been difficult if we had chosen to mail questions or used an internet-based survey monkey. Such efforts in the past have not yielded adequate results. We used a structured, self-administered questionnaire on a cross-section of members and associate members attending a recent National Nephrology Association meeting. This questionnaire was developed by the authors, taking into considerations the relevant information we sought to gather to explore the challenges and possible solutions to peritoneal dialysis in Nigeria. The main questions of interest included the professional status of respondents, history of use of PD in their centres, training and skills in use of PD, challenges of PD, and re-introduction of PD. The questionnaire was in English language and was pre-tested. A total of 150 questionnaires were distributed to participants who included consultant nephrologists, nephrology trainees, nephrology nurses and nephrology technicians. The questionnaires were anonymized and carried only serial numbers. The questionnaires had multiple responses sections, but as is common in self-administered questionnaires there were incomplete responses as well. Data was first entered into an Excel spread sheet, and then imported into Statistical Package for Social Sciences (SPSS). Data analysis was done with SPSS version 20 [10]. Group responses were calculated as percentages for comparisons.

## Results

A total of 125 questionnaires were returned. There were 68(54.4%) doctors, 43(27.2%) nurses, and 14(11.2%) renal technicians, all from medical institutions with renal treatment programs who participated in the study (Table 1). In centres where peritoneal dialysis had been used, the common types of peritoneal dialysis were intermittent rigid catheter, intermittent flexible catheter, and CAPD in almost equal proportions, 16%, 19.2% and 17.6% respectively. CCPD has been least utilized as a modality of peritoneal dialysis (8.0%) (Table 2 and Table 3). Majority of the centres were established more than 10 years ago. Fifty-eight (46.4%) respondents indicated that PD had been in use in their centres for renal failure treatment, while the rest claimed it had never been used. The use of PD had ceased in these centres for periods ranging from less than 5 years to more than 10 years (Table 4).

**Table 1:** Professional status of respondents (N=125)

Status	N	%
Doctors (n=68) Consultants	24	19.2
Nephrology trainees	44	35.2
Nephrology nurses	43	34.4
Nephrology technicians	14	11.2
**Total**	125	100

**Table 2:** Individual experience with PD use in 125 respondents*

Type of PD use	N	%
Intermittent Rigid Catheter	20	16.0
Intermittent Flexible Catheter	24	19.2
CAPD	22	17.6
CCPD	10	8.0

* Multiple responses and no response

**Table 3:** Problems most encountered with PD use (58 respondents)

Problems	N	%
Financial constraints	30	51.7
Inadequate fluid supply	29	50.0
Frequent line blockage	13	22.4
Frequent Infections	10	17.2
Frequent fluid leakage	5	8.6
Haemorrhage	1	1.7

* Multiple responses and no response

**Table 4:** When PD was first used and reasons for stopping PD use (57 respondents)

PD last used, years (N=57)	n	%
<5	1	2
6-10	3	5
>10	53	93
Reasons for stopping PD*		
Lack of PD catheters	13	22.8
Lack of PD fluids	29	50.8
Lack of expert personnel	9	15.8
Lack of satisfactory results	6	10.6

* Multiple responses and no response

The most common problems encountered with PD use are financial constraints (51.7%), inadequate fluid supply (50%), frequent line blockage (22.4%) and frequent infections (17.2%). Reasons attributed to the stoppage of PD in the centres included lack of PD fluids (50.8%), unavailability of PD catheters (22.8%), lack of expert personnel to train (15.8%). Sixty-five percent of consultant nephrologists strongly agree that peritoneal dialysis training should be mandatory in contrast to 30.6% of nephrology nurses and 27.9% of nephrology trainees, who nonetheless mostly agree that PD should be made mandatory (Table 5). Consultant nephrologists are more likely to see peritoneal dialysis as the only viable option for dialysis than nephrology trainees, nurses and technicians (Table 5).

**Table 5:** Mandatory option and viability of peritoneal dialysis use

Should peritoneal dialysis be made mandatory?					
Status	Strongly agree n (%)	Agree n (%)	Don't agree n (%)	Indifferent n (%)	Total N
Consultant nephrologists	15(65.2)	5(21.7)	2(8.7)	1(4.3)	23
nephrology trainees	12(27.9)	24(58.8)	6(14.0)	1(2.3)	43
Nephrology nurses	11(30.6)	19(52.8)	5(13.9)	1(2.8)	36
Nephrology technicians	2(20.0)	5(50.0)	1(10.0)	2(20.0)	10
Do you see peritoneal dialysis as the only viable option?					
consultant nephrologist	8(38.1)	5(23.8)	6(28.6)	2(9.5)	21
nephrology trainees	7(24.0)	11(32.0)	11(36.0)	4(8.0)	33
Nephrology nurses	5(19.2)	5(19.2)	9(34.6)	7(26.9)	26
Nephrology technicians	(0(0.0)	1(12.5)	3(37.5)	4(50.0)	8

* There were no responses in some cases

## Discussion

The use of peritoneal dialysis or indeed any form of dialysis will depend to a large extent on the advocacy, knowledge, and willingness to adopt it by those at the forefront of renal care: doctors, nurses and technicians. They are also the ones who will be in a vantage position to overcome barriers militating against its use [11, 12] Of course, patients' satisfaction, perception, and preference are also very important [13, 14]. This is the rationale for engaging these professionals. Nephrology nurses were in the majority of those interviewed and this reflects their proportion in the membership of the association where they number more than two thirds of about 700 members. Their views also count enormously because they are more in contact with patients than any other health professionals. They are also involved in the training of patients and showing empathy in their interaction with the patients [15].

The first mode of renal replacement therapy in Nigeria was peritoneal dialysis [1], and yet over the years this seems to have been replaced by haemodialysis. Over the years the uptake of patients on peritoneal dialysis programmes has waned. In our survey, 53% of respondents claimed that peritoneal dialysis has not been used for more than ten years. This indeed is the general trend globally, and especially in Africa [2, 16, 17], where haemodialysis has replaced peritoneal dialysis. Reasons adduced for these include high occurrence of infections, unavailability of peritoneal fluids, and lack of marketers for peritoneal dialysis consumables.

With regards to the patient, as in previous reports, the two most common complications are frequent line blockage and peritonitis [3, 18, 19]. Peritonitis adds to the cost of treatment because patients have to be treated for this; it prejudices future treatment with PD and could also be life-threatening. It seems then that most centres were able to cope with this, because when asked specifically the reasons for stopping PD the commonest reasons were lack of fluids (50.8%) and lack of catheters (22.8%). Until about fifteen years ago, there was a company in Nigeria that produced PD fluids, and this was the major source of supply for most centres in Nigeria. With the economic downturn and the decline in the manufacturing sector, this company folded up. In addition, as in other parts of the world [16, 17], there was an increase in the number of haemodialysis centres in Nigeria, and this has indirectly increased the cost of renal replacement therapy in Nigeria [20]

Majority of all four categories of nephrology professionals at least agree that PD should be made mandatory. There is an increasing advocacy to make this the first choice of renal replacement therapy [21-23], because of better survival, flexibility, and lower cost [21]. Thirty-eight percent of consultants strongly agree that PD is the only viable option, while at least thirty three percent of nephrology trainees and nurses don't agree that PD is the only viable option. This may reflect a generation gap in practice. Moreover, nephrology consultants, the most experienced in the practice, in strongly agreeing that PD is the only viable option may also have hinted at the need for more training and uptake of patients into the programme, as concluded in a recent review [24].

## Conclusion

Peritoneal dialysis in Nigeria, though rapidly declining in its use, is still perceived as an important option for renal failure treatment, and efforts at its re-integration should be actively pursued. Main obstacles to its use are availability and cost of consumables. Efforts to overcome the factors militating against its use should be positively pursued so that peritoneal dialysis will be re-integrated into the mainstream of renal replacement therapy once more. These will include training and collaboration with all stakeholders, including health authorities.

### What is known about this topic

- Peritoneal dialysis is a useful mode of renal replacement therapy;
- It was once popular in earlier days of nephrology practice in Nigeria;
- Many centres in Nigeria no longer offer peritoneal dialysis.

### What this study adds

- Consultant nephrologists, trainees, nephrology nurses and technicians have limited experience with use of peritoneal dialysis in Nigeria;
- Lack of consumables, and not only infection, is a major reason for the dwindling use of peritoneal dialysis;
- Efforts at resuscitating the use of peritoneal dialysis are welcome by practitioners in nephrology.

## Competing interests

The authors declare no competing interests.
